# Machine Learning Assistants Construct Oxidative Stress-Related Gene Signature and Discover Potential Therapy Targets for Acute Myeloid Leukemia

**DOI:** 10.1155/2022/1507690

**Published:** 2022-08-22

**Authors:** Jinhua Zhang, Zhenfan Chen, Fang Wang, Yangbo Xi, Yihan Hu, Jun Guo

**Affiliations:** ^1^The First Clinical Medical College of Jinan University, First Affiliated Hospital of Jinan University, Guangzhou 510630, China; ^2^School of Life Science, Northwestern Polytechnical University, Xian, 710072, China; ^3^Department of Hematology and Immunology, Myeloma Center Brussels, Vrije Universiteit Brussel, Brussels B-1090, Belgium

## Abstract

**Background:**

Oxidative stress (OS) is associated with the development of acute myeloid leukemia (AML). However, there is lack of relevant research to confirm that OS-related genes can guide patients in risk stratification and predict their survival probability.

**Method:**

First, we Data from three public databases, respectively. Then, we use batch univariate Cox regression and machine learning to select important characteristic genes; next, we build the model and use receiver operating characteristic curve (ROC) to evaluate the accuracy. Moreover, GSEAs were performed to discover the molecular mechanism and conduct nomogram visualization. In addition, the relative importance value was used to identify the hub gene, and GSE9476 was to validate hub gene difference expression. Finally, we use symptom mapping to predict the candidate herbs, targeting the hub gene, and put these candidate herbs into Traditional Chinese Medicine Systems Pharmacology (TCMSP) to identify the main small molecular ingredients and then docking hub proteins with this small molecular.

**Results:**

A total of 313 candidate oxidative stress-related genes could affect patients' outcomes and machine learning to select six potential genes to construct a gene signature model to predict the overall survival (OS) of AML patients. Patients in a high group will obtain a short survival time when compared with the low-risk group (HR = 3.97, 95% CI: 2.48-6.36; *p* < 0.001). ROC results demonstrate the model has better prediction efficiency with AUC 0.873. GSEA suggests that this gene is enriched in several important signaling pathways. Nomogram is constructed and is robust. PLA2G4A is a hub gene of signature and associated with prognosis, and Nobiletin could target PLA2G4A for therapy AML.

**Conclusion:**

We use two different machine learning methods to build six oxidative stress-related gene signatures that could assist clinical decisions and identify PLA2G4A as a potential biomarker for AML. Nobiletin, targeting PLA2G4, may provide a third pathway for therapy AML.

## 1. Introduction

Acute myeloid leukemia (AML) was derived from abnormal stem cell precursors of the myeloid lineage [1]. These proliferative clonal hematopoietic precursor cells damage the normal hematopoiesis and cause a series of clinical symptoms. Although AML is a rare disease compared with other newly diagnosed cancers, it accounts for more than 15% of acute leukemia with more than 30% mortality [2, 3]. Currently, molecular and cytogenetic features are recognised as key prognostic factors for the disease diagnosis. Hematopoietic stem cell transplantation (HSCT) remains the only way to cure this disease, but the overall survival (OS) is still stagnant due to severe infection and acute graft-versus-host disease (aGVHD) after transplantation [3, 4]. More than 70% of patients receiving conventional chemotherapy will eventually relapse or become refractory leukemia [5]. Thus, it is of great importance to gain an understanding of the genetic variations in this disease and explore new targets for predicting prognosis and direct treatment.

Oxidative stress (OS) refers to a state of imbalance between reactive oxygen species (ROS) and antioxidant effects in the body, which is characterized as inflammatory infiltration of neutrophils, increased secretion of proteases, and the production of a large number of oxidative compounds [6]. ROS have been confirmed to be associated with cellular signaling and gene expression in the normal cellular process [7, 8]. However, once the endogenous ROS is not adequately eliminated by the antioxidant system, the prooxidant/antioxidant balance is lost and leads to the occurrence of OS, which will damage the biological processes and the DNA repair mechanisms, leading to kinds of diseases and carcinogenesis, such as neurological disease, cardiovascular disease, breast cancer, prostate cancer, and hematologic malignancies [9–14]. On the other hand, ROS can undertake an opposite role in tumor therapy by inducing cell apoptosis [14]. Therefore, clarifying the dual role of OS in the pathogenesis and treatment of AML and selecting the potential beneficial population would lay the foundation for individualized precision treatment.

In the present work, we use two different machine learning methods to select candidate prognosis genes in oxidative stress-related gene sets and build a six-gene signature model to predict the AML patients' outcomes. In addition, we also identify PLA2G4A as a hub gene of signature and associated with prognosis and found that Nobiletin, a type of traditional Chinese medicine, targeting PLA2G4 may provide a third pathway for therapy AML.

## 2. Methods and Materials

### 2.1. Data Obtaining and Prepared

Public datasets were applied to this study: The Cancer Genome Atlas Program (TCGA), Genotype-Tissue Expression (GTEx), and Gene Expression Omnibus (GEO), respectively. We obtain AML data from TCGA database, which includes RNAseq records and clinical information of patients. Original count data are transferred to TPM style and extract 1399 oxidative stress-related genes, recorded by gene card database, from the expression profile to construct a new oxidative stress-related gene matrix for model building. Overall survival (OS) was defined as the endpoint. RNAseq data of donor bone marrow are from the GTEx database. GSE9476, including 38 donors and 26 AML samples, was used to validate hub gene differential expression between healthy hematopoietic cells and leukemic blasts. The 3D structure of hub protein and small-molecule structures are sourced from PDB and Pub Chem, respectively.

### 2.2. Batch Univariate Cox Regression to Identify Prognosis-Related Genes

Too many genes will affect patients' outcomes; here, we use batch univariate Cox regression to identify prognosis-related genes of oxidative stress. After analysis, genes with a *p* value less than 0.05 were identified as significant factors, and this gene will be input into the next model to perform dimensionality reduction.

### 2.3. Machine Learning to Select Important Characteristic Genes

Random forest and lasso regression are performed to identify important characteristic genes of oxidative stress. The importance of the random forest algorithm was defined as 0.3, and then, all of these requirement genes were put into the lasso regression model, which model set significant criterion was lambda is minimal.

### 2.4. Predictive Model Construct and Validation

For building the final prediction model, we use multivariate Cox regression to analyze the significant oxidative stress-related genes; as above standard, the *p* value is also set as less than 0.05. After selecting all of these requirement genes, we build the final model for the prediction of patient outcome, according to the regression coefficients. Each patient will obtain one risk score, patients will be divided into high- and low-risk groups, according to the median value. Survival analyses were conducted by log-rank test. Forty percent of the total data were set as a test dataset to validate robust of the above model. Area under ROC was used to evaluate the predictive accuracy.

### 2.5. Difference Expression Genes between High-Risk and Low-Risk Groups

To identify the differential expression genes between the high-risk and low-risk groups, the limma package was used to conduct this procedure, and the criteria of significant genes were set as absolute of log fold change more than 2 and *p* value less than 0.05.

### 2.6. GSEA of Differential Expression Genes

The molecular mechanism of differential expression genes between the high-risk and low-risk groups is unclear; here, we use the GSEA function, which provides by the cluster profile package, to do GO and KEGG pathway enrichment analysis. *p* value less than 0.05 was identified as a significant enrichment result.

### 2.7. Nomogram Construction and Evaluation

Nomogram is more than eyes and provides a convenient tool for clinical physicians to assist clinical decisions. Significant genes from the above multivariate Cox regression results will be considered and put into the VRPM package to conduct visualization. The area under the curve (AUC) of receiver operating characteristic (ROC) and calibration curve were used to evaluate the model's robustness.

### 2.8. Hub Gene Selection and Validation

In the gene panel, not all the genes play an important role in the model, so we select the importance value of the model gene and order it from high to low, and the biggest value of the important gene was identified as the hub gene. Donor patients' bone marrow, which obtains from the GTEx database, will be compared with AML patients' bone marrow to validate hub gene difference expression. In addition, sample from GSE9476 also repeats the above operation. Moreover, survival analysis was performed to conduct by survminer package, to compare survival probability difference between high and low expression patients.

### 2.9. Screening Candidate Herbs Targeting Hub Protein

Traditional Chinese medicine has been confirmed that has the potential ability to target tumor markers. We use a symptom mapping (symMap Version 2.0) database to predict the candidate herbs, which will target the hub gene. Herbs with FDR less than 0.05 will be selected. The top 10 requirement herbs will be selected and also checked by the previous literature, which has been reported to have the ability of anticancer function for preparing docking with hub proteins. Candidate herbs will be input into Traditional Chinese Medicine Systems Pharmacology (TCMSP) to identify the main small molecular ingredients, according to oral bioavailability (OB) more than 30 and drug-likeliness (DL) more than 0.18.

### 2.10. Docking Structures between Proteins and Small Molecular Drugs

Before docking both structures, we need prepared ligand and receptor structures. So, we download the hub protein's 3D structure from the protein data bank (PDB; https://www.rcsb.org/) and obtain small molecular drug structure form Pub Chem database (https://pubchem.ncbi.nlm.nih.gov/), respectively. Then, an online tool will conduct a docking program (https://cadd.labshare.cn/cb-dock2/php/index.php).

## 3. Results

### 3.1. Prognosis Genes in Oxidative Stress-Related Gene Set of AML

We use a batch univariate regression model to filter no significant genes, which could not affect patients' outcomes, and some prognosis-related genes in the oxidative stress-related gene set were selected. The results of the batch univariate regression model show that a total of 313 candidates' oxidative stress-related genes could affect patients' outcomes (Supplement Table 1).

### 3.2. Machine Learning to Select Candidate Model Genes

**Figure 1:**
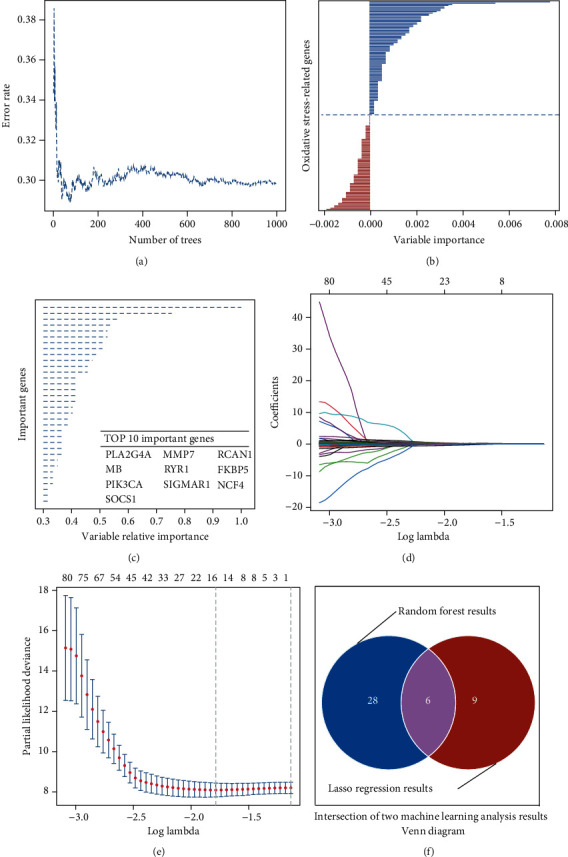
Machine learning to identify important oxidative stress-related genes for the prognosis model. (a, b) The error rate of the random forest model is 29.83%, and the better and poor prognosis genes are ordered by their importance. (c) Thirty-four prognosis-related genes are important more than 0.3. (d, e) Fifteen candidate genes are extracted from the expression profile by the lasso regression model. (f) Six potential genes were identified after the merge of the results of the above two different machine learning algorithms.

Random forest and lasso regression models were used to select candidate model genes. On the one hand, we put the expression matrix to the random forest model. The results show that the error rate of the random forest model is 29.83%, and the better and poor prognosis genes are ordered by importance (Figures 1(a) and 1(b)). When we set variable relative importance to more than 0.3, 34 prognosis-related genes are selected, and the top 10 significant are shown in Figure 1(c) (Supplement Table 2). On the other hand, the above expression matrix is also put into the lasso regression model, when the model selects the minimal lambda value; 15 candidate genes are extracted from the expression profile (Figures 1(d) and 1(e)). Then, we merge the results of the above two different machine learning algorithms. Six potential genes were identified to construct a gene signature model (Figure 1(f)).

### 3.3. Six-Gene Signature Could Predict the OS of AML Patients

**Figure 2:**
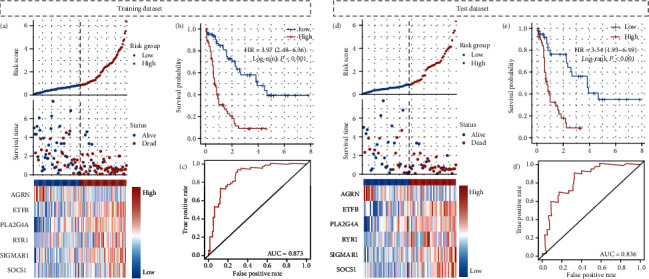
Six oxidative stress-related gene signature construction and internal validation. (a) Each patient will obtain a risk score, which is calculated by the model formula. (b) Patients in a high group will obtain a short survival time when compared with the low-risk group, and this difference is significant (HR = 3.97, 95% CI: 2.48-6.36; *p* < 0.001). (c) ROC demonstrates the model has better prediction efficiency with AUC is 0.873. (d–f) Internal validation results also support the above conclusions, and the AUC is 0.836.

After multivariate Cox regression model analysis, we find six genes from machine learning methods are included in the model (Table 1). So, in the next step, we build a six-gene signature model to predict the OS of AML patients, according to the coefficient of multivariate Cox regression. After building the signature model, every patient will obtain a risk score, which calculated by the model formula, risk score = (−0.391) × AGRN + (0.827) × ETFB + (0.236) × PLA2G4A + (0.650) × RYR1 + (0.404) × SIGMAR1 + (0.473) × SOCS1. After the count, patients will be divided into low-risk and high-risk groups, based on median value (Figure 2(a)). Figure 2(b) shows that patients in the high group will obtain a short survival time when compared with the low-risk group, and this difference is significant (HR = 3.97, 95% CI: 2.48-6.36; *p* < 0.001). We use ROC to evaluate the prediction accuracy of the model, and the results demonstrate the model has better prediction efficiency with AUC is 0.873 (Figure 2(c)). Internal validation results also support the above conclusions. Patients with low-risk scores mean longer living times when compared with high-risk score patients. In addition, the ROC of the validation dataset also shows the model could predict patients' outcome accuracy with AUC equal to 0.836 (Figures 2(d)–2(f)).

### 3.4. Six-Gene Signature with Clinical Factors

**Figure 3:**
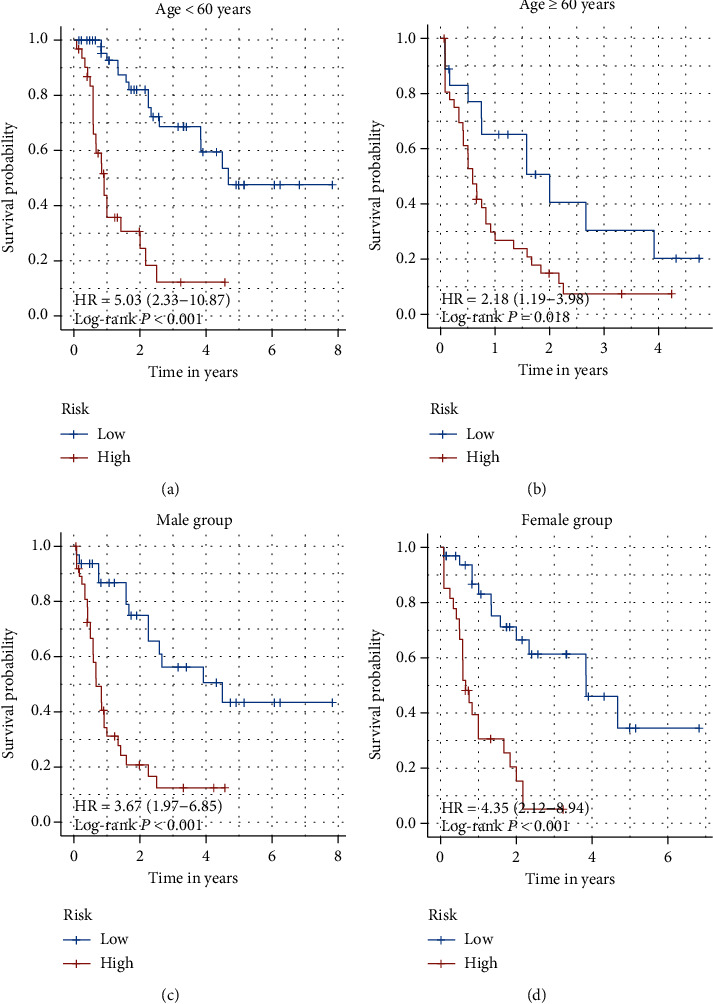
Signature with clinical variables. (a) In the age subgroup, patients under 60 years old, the high-risk score means shorter survival time, when compared with the low-risk group (HR = 5.03, 95% CI: 2.33-10.87; *p* < 0.001). (b) This conclusion also confirmed by patients more than 60 years old (HR = 2.18, 95% CI: 1.19-3.98; *p* = 0.018). (c, d) As for the sex subgroup, patients with low-risk scores always represent a better prognosis, when compared with a high-risk score, no matter which sex they are (HR = 3.67, 95% CI: 1.97-6.85; *p* < 0.001 vs. HR = 4.35, 95% CI: 2.12-8.94; *p* < 0.001, respectively).

Age and sex are both important clinical characteristics for AML patients. Here, we perform survival analysis to discover the difference between clinical subgroups. In the age subgroup, we find that in patients under 60 years old, the high-risk score means a shorter survival time, when compared with the low-risk group (HR = 5.03, 95% CI: 2.33-10.87; *p* < 0.001) (Figure 3(a)). This conclusion also confirmed by patients more than 60 years old (HR = 2.18, 95% CI: 1.19-3.98; *p* = 0.018) (Figure 3(b)). As for the sex subgroup, patients with low-risk scores always represent have a better prognosis, no matter which sex they are (HR = 3.67, 95% CI: 1.97-6.85; *p* < 0.001 vs. HR = 4.35, 95% CI: 2.12-8.94; *p* < 0.001, respectively) (Figures 3(c) and 3(d)).

### 3.5. Different Expression Genes and Enrichment Analysis

**Figure 4:**
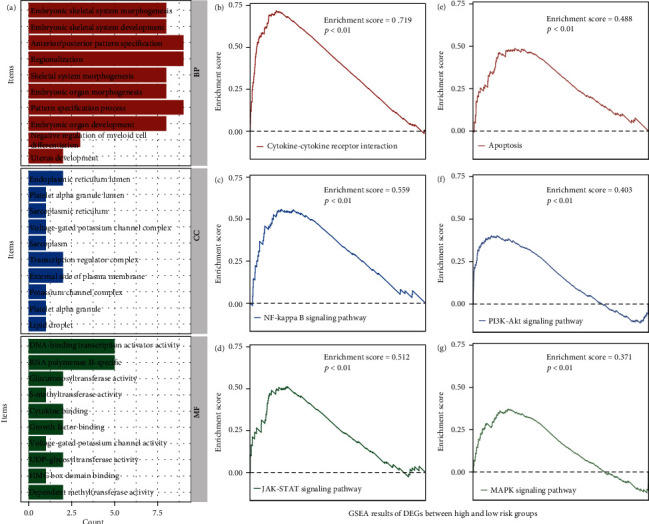
GSEA results of differential expression genes. (a) GSEA shows that this gene is enriched in embryonic skeletal system morphogenesis, endoplasmic reticulum lumen, and RNA polymerase II-specific, and the (b–g) involved pathways are cytokine-cytokine receptor interaction pathway, NF-kappa B signaling pathway, JAK-STAT signaling pathway, apoptosis, PI3K-AKT signaling pathway, and MAPK signaling pathway.

Patients with different risk scores have different prognoses. Identifying different expression genes between two groups is good to discover the molecular mechanism in the future. The different expression analyses demonstrate a total of 49 different expression genes between low- and high-risk groups (Supplement Table 3). After doing GSEA, we found that this gene is enriched in embryonic skeletal system morphogenesis, endoplasmic reticulum lumen, and RNA polymerase II-specific (Figure 4(a)), and the involved pathways are cytokine-cytokine receptor interaction pathway, NF-kappa B, PI3K-AKT, and MAPK signaling pathway (Figures 4(b)–4(g)).

### 3.6. Nomogram Is a Useful Tool for Assistant Clinical Decision

**Figure 5:**
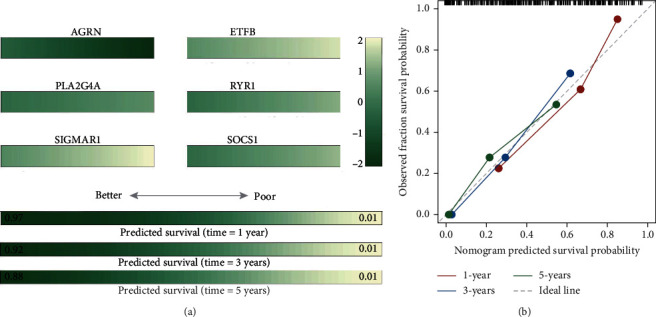
Nomogram for six model genes. (a) Nomogram, based on six-gene expression, to an assistant clinical decision, according to six-gene expression, patients will obtain six score values and accumulate six values to become one total score and projection onto the survival axis to obtain patients' 1-year, 3-year, and 5-year survival probability, respectively. (b) Calibration curve analysis results suggest that survival prediction probability was close to the ideal line.

We have demonstrated that six gene signatures could predict patients' outcome accuracy, so we build a nomogram, based on six gene expressions, to assist clinical decisions. This model is shown in Figure 5(a), and according to six gene expressions, patients will obtain six score values and accumulate six values to become one total score and projection onto the survival axis to obtain patients' 1-year, 3-year, and 5-year survival probability, respectively. The ROC of the model is 0.761, and the C index of this nomogram is 0.774. Calibration curve analysis results suggest that survival prediction results of 1-year, 3 year, and 5-year survival probability were close to the ideal line (Figure 5(b)).

### 3.7. PLA2G4A Is a Hub Gene of Signature and Associated with Prognosis

**Figure 6:**
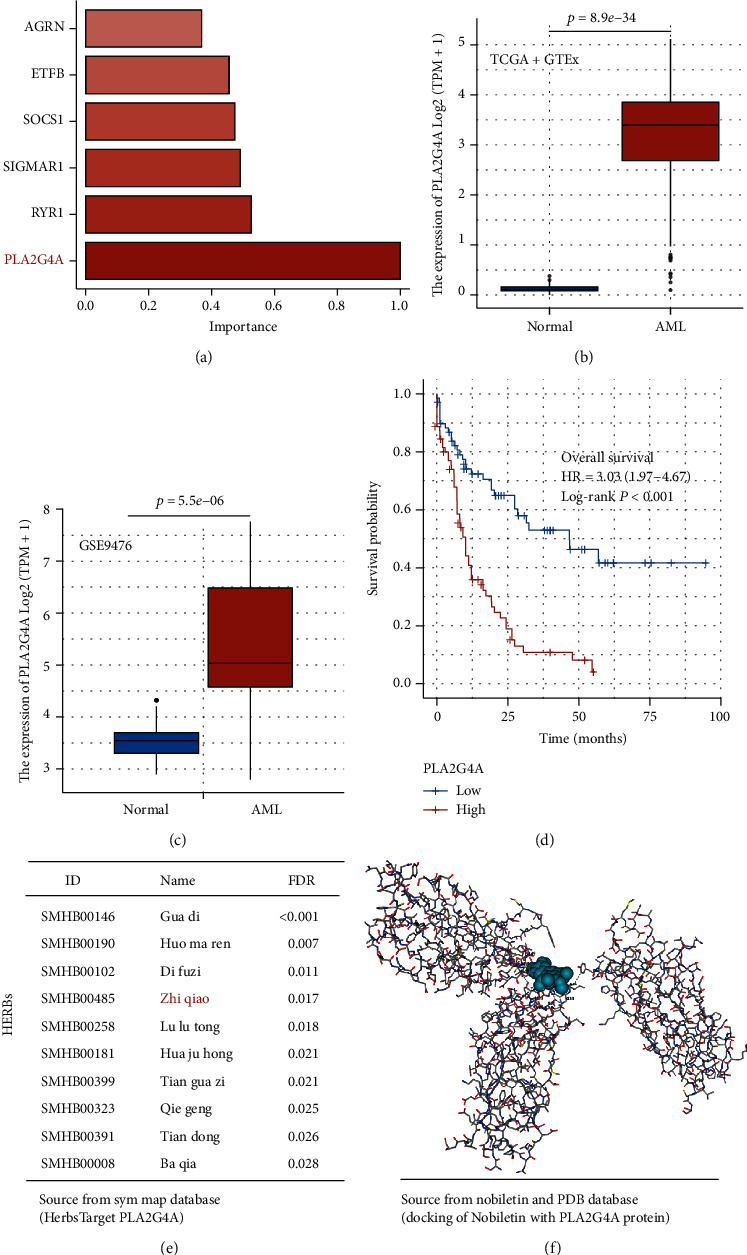
PLA2G4A is a candidate therapeutic target for AML. (a) PLA2G4A has the biggest value of relative importance in six model genes, and (b, c) it is a high expression in leukemic blasts when compared with healthy hematopoietic cells (*p* = 8.9*e* − 34 vs. *p* = 5.5*e* − 06, respectively). (d) High expression of this gene will lead to a poor outcome when compared with low expression patients (HR = 3.03, 95% CI: 1.97-4.67; *p* < 0.001). (e) The top 10 herbs target PLA2G4A; (f) docking Nobiletin, the main components of this Zhiqiao, with PLA2G4A protein.

We extract the relative importance of six model genes, and the PLA2G4A has the biggest value, so it was confirmed as the model hub gene (Figure 6(a)); to validate the potential value, we use external data set, GTEx, and GSE9476, to observe the expression difference between donor bone marrow and leukemic blasts from AML patients. The result demonstrates that PLA2G4 is a high expression in leukemic blasts and low expression in healthy hematopoietic cells (*p* = 8.9*e* − 34 vs. *p* = 5.5*e* − 06, respectively) (Figures 6(b) and 6(c)). Survival analysis also shows that high expression of this gene will lead to a poor outcome when compared with low expression patients (HR = 3.03, 95% CI: 1.97-4.67; *p* < 0.001) (Figure 6(d)).

### 3.8. Nobiletin Targeting PLA2G4A Provides a Third Pathway for Therapy AML

A total of 41 required herbs had been predicted, and the top 10 herbs are listed in Figure 6(e) and Supplement Table 4. Of these ten herbs, Zhiqiao has been reported to have a potential function as an anticancer. To identify which ingredients are important for these herbs, we input in into the TCMSP database. The results show that Hesperetin, Nobiletin, Naringenin, Marmin, and Beta-sitosterol are the main components of this drug (Table 2). During these components, Nobiletin has the best OB (61.67%) and highest DL (0.52); it was selected as a candidate small molecular drug to target PLA2G4A. The docking results also demonstrate the above conclusion (Figure 6(f)).

## 4. Discussion

Although advances have been achieved in the therapeutic options which greatly improve the overall response rate, the long-term prognosis of this disease remains dismal, especially among elder patients [15]. Besides, the complex molecular and cytogenetic abnormalities make AML a kind of heterogeneous disease with differential prognosis even in the same risk group by clinical practice guidelines [16]. All these revealed that insight into the genetic landscape of AML would benefit more patients. The development of various sequencing technologies has provided more information on the mechanism of pathogenesis, chemoresistance, and more refined prognostic stratification of AML in the past decades. Recently, Mer et al. [15] proposed a unique subtype of NPM1-mutated AML with different biological and therapeutical implications based on a stem cell signature. A set of mitochondrial metabolism proteins was also identified as potential targets associated with leukemia progression by multiomics [17]. Furthermore, some gene-based signatures have been constructed to predict the prognosis of AML as in other cancers [18, 19]. All these have brought new opportunities for the treatment of AML.

The maintenance of the quiescent state of hematopoietic stem cells (HSCs) depends on a condition of anaerobic glycolysis with low ROS generation, while compelling evidence has indicated that leukemia stem cells (LSCs), which are considered the main part of drug resistance, are more dependent on oxidative respiration with high ROS levels companied by an imbalance of oxidative and antioxidant, which promote the progression of leukemia by activating the pathways involved in the cell proliferation, survival, and invasion [20, 21]. Previous studies have proved that the redundant ROS could be a risk factor for tumorigenesis and the drug resistance role of ROS in varied leukemia modes [22, 23]. Interestingly, LSCs are more susceptible to external antioxidants, and ROS and lipid peroxidation by-products can trigger cell apoptosis, which also brings new chemotherapy options. These all suggest a bidirectional role of ROS in leukemia [24]. Given the above complex mechanism, we aim to construct an accurate model which can provide precise predictivity and guide stratification therapy for clinical application.

As shown in our work, we used machine learning to select six hub OS-related genes, which demonstrated robust predictive ability in AML populations. Agrin (AGRN) has been described as a multifunctional heparan sulfate proteoglycan, which can regulate angiogenesis and has a board-ranging impact on the tumor microenvironment (TME) in HCC and papillary thyroid carcinoma (PTC) [25, 26]. However, nothing has been reported on the exact function of AGRN in AML. Electron transfer flavoprotein *β* subunit (ETFB) can transfer the electrons to the electron transport chain (ETC) and maintain the generation of ATP. Caplan et al. [17] recently found that increased mitochondrial stress and apoptosis in AML mouse models can be induced by silencing ETFB, which suggests that ETFB could be a potential therapeutic target for AML. Moreover, the placental phospholipase A24A (PLA2G4A), sigma 1 receptor (Sig1R), and suppressors of cytokine signaling 1 (SOCS1) have been reported in varied diseases which are involved in the stress response biological procedure [27–29]. However, to the best of our knowledge, abscisic acid receptor (PYR1) has been shown as a stomatal regulation response to drought stress in plants, and no studies regarding abscisic acid receptor (PYR1) have been reported in human diseases or cancers [30]. Among these six genes, PLA2G4A has the most important in our model, which is one of the cytosolic placental phospholipases A2 (cPLA2) family and can catalyze the hydrolysis of membrane phospholipids to release arachidonic acid (AA) and lysophospholipid. It has been identified as an index in response to oxidative stress in preeclampsia and might be due to the oxidation of AA [27]. Higher expression of PLA2G4A is positively correlated with the migration and invasion of lung cancer cells and unfavorable prognosis in breast cancers [31, 32]. Previous studies also revealed that PLA2G4A expression could be an independent diagnostic and prognostic marker in patients with non-M3/NPM1 WT AML patients [33], which was also confirmed in our study. Nevertheless, whether this differential prognosis is caused by PLA2G4A through oxidative stress remains to be further investigated.

To explore the specific mechanism of OS-related genes in AML, we carried out functional annotation of DEGs between the high and low gene expression groups. The OS-related genes were enriched in the cytokine-cytokine receptor interaction pathway, NF-kappa B signaling pathway, JAK-STAT signaling pathway, apoptosis, PI3K-AKT signaling pathway, and MAPK signaling pathway as revealed by the GSEA results, which have been identified by previous studies serving as an important role in the pathogenesis and progression of AML. The PI3K/AKT pathway was proved to play important roles in regulating cell proliferation, differentiation, apoptosis, and migration in kinds of human diseases and cancers, such as diabetes, colorectal cancer, and AML [34, 35]. Some scholars also reported the PI3K/AKT pathway is associated with oxidative stress-mediated survival of melanoma and when targeting the PI3K/AKT and MAPK/ERK signaling pathway exerts an anticancer effect in leukemia cells by induction of oxidative stress and the cellular antioxidant defense mechanisms, which suggest PI3K/AKT and MAPK/ERK signaling pathway might involve in the leukemia cell apoptosis caused by oxidative stress [36, 37].

However, there are some limitations to our study. First, we did not distinguish between OS-related genes that promote leukemia proliferation and invasion with genes that mediate leukemia cell apoptosis via chemotherapy-induced OS. More datasets with pre/posttreatment information need to be included to clarify this bidirectional effect of OS. Second, our results need to be validated in a clinical trial in the further.

## 5. Conclusion

Our six oxidative stress-related gene signatures could predict AML patients' outcome accuracy, and this model is robust. It may become a useful tool to assist clinical decisions. In addition, we identify PLA2G4A as a potential biomarker for AML. Nobiletin, targeting PLA2G4, may provide a third pathway for therapy AML.

## Figures and Tables

**Table 1:** Multivariate Cox regression for model genes.

Gene symbol	Coef	HR	*p* value	95% CI	
				Lower	Upper
AGRN	-0.391	0.676	0.507	0.902	0.008
ETFB	0.827	2.289	1.051	4.985	0.037
PLA2G4A	0.236	1.266	0.965	1.661	0.088
RYR1	0.650	1.917	1.239	2.966	0.003
SIGMAR1	0.404	1.498	1.026	2.187	0.036
SOCS1	0.473	1.605	1.177	2.189	0.003

**Table 2:** Ingredients of Chinese traditional medicine Zhiqiao.

Mol ID	Molecule name	OB (%)	DL	MW	A log*p*	FASA-	HL
MOL002341	Hesperetin	70.31	0.27	302.3	2.28	0.33	15.78
MOL005828	Nobiletin	61.67	0.52	402.43	3.04	0.13	16.2
MOL004328	Naringenin	59.29	0.21	272.27	2.3	0.4	16.98
MOL013381	Marmin	38.23	0.31	332.43	3.11	0.31	4.68
MOL000358	Beta-sitosterol	36.91	0.75	414.79	8.08	0.23	5.36

## Data Availability

All data used in the study are available in The Cancer Genome Atlas (TCGA) and Gene Expression Omnibus (GEO) databases.

## Supplementary Materials

Supplementary Materials Supplement Table 1: prognosis-related oxidative stress genes by filter batch univariate Cox regression. Supplement Table 2: genes with a relative importance of more than 0.3 in the random forest model. Supplement Table 3: different expression genes between low- and high-risk groups. Supplement Table 4: candidate herbs targeting PLA2G4A protein.
