# Supplementary material for: Machine Learning Assistants Construct Oxidative Stress-Related Gene Signature and Discover Potential Therapy Targets for Acute Myeloid Leukemia
Source: Oxid Med Cell Longev. 2022 Aug 22;2022:1507690. doi: 10.1155/2022/1507690 (PMC9423988; doi:10.1155/2022/1507690)
Supplement: Supplementary Materials — Supplement Table 1: prognosis-related oxidative stress genes by filter batch univariate Cox regression. Supplement Table 2: genes with a relative importance of more than 0.3 in the random forest model. Supplement Table 3: different expression genes between low- and high-risk groups. Supplement Table 4: candidate herbs targeting PLA2G4A protein. [file 1507690.f1.zip › 1507690.f1.pdf]

gene	HR	z	pvalue	lower	upper
IL2RA	1.3803414	4.5565253	5.20E-06	1.2016337	1.5856267
PTPA	3.8700162	4.5386555	5.66E-06	2.1573274	6.9423978
ETFB	4.7694366	4.5267156	5.99E-06	2.4249702	9.3805383
ENO1	2.7003107	4.3564179	1.32E-05	1.7271082	4.2218999
G6PD	2.0079786	4.3535028	1.34E-05	1.4670866	2.748289
SDHA	3.9155582	4.3500792	1.36E-05	2.1169257	7.2423873
UCP2	2.0453867	4.336908	1.45E-05	1.4802251	2.8263315
AKAP9	0.4775793	-4.329503	1.49E-05	0.3417823	0.6673312
RAC2	2.4219889	4.3124111	1.61E-05	1.6201988	3.6205621
SIGMAR1	2.0236532	4.3090199	1.64E-05	1.4685531	2.7885764
PLA2G4A	1.6840563	4.2865654	1.81E-05	1.3269625	2.1372463
TFEB	1.7179262	4.282297	1.85E-05	1.3410511	2.2007143
CHCHD10	1.9523442	4.253982	2.10E-05	1.4344536	2.6572123
TGFB1	2.0397667	4.2046443	2.61E-05	1.4630926	2.8437353
LPO	0.7445387	-4.200877	2.66E-05	0.6488061	0.8543968
SOCS1	1.8231145	4.1898492	2.79E-05	1.3766012	2.4144585
CSK	2.5256893	4.1683553	3.07E-05	1.633731	3.9046246
ATP13A2	1.9475583	4.1006767	4.12E-05	1.4162023	2.678278
IRAK1	2.4785175	4.0866786	4.38E-05	1.6037556	3.8304146
DAXX	6.4445556	4.035664	5.44E-05	2.6073518	15.928919
HGF	0.7686149	-4.015919	5.92E-05	0.6759726	0.8739538
PTPN1	3.0297183	4.0126071	6.01E-05	1.7630336	5.2064763
MLYCD	0.2776846	-4.009278	6.09E-05	0.1484322	0.5194879
RYR1	2.3956574	3.997977	6.39E-05	1.5610469	3.6764907
TXN2	3.6020703	3.9371596	8.25E-05	1.903242	6.8172677
NDUFS2	5.5598688	3.9312615	8.45E-05	2.3637757	13.077443
PPIF	2.0521932	3.91106	9.19E-05	1.4313807	2.9422619
SDHB	2.9948476	3.8123846	0.0001376	1.7039951	5.2635784
MPO	0.8744513	-3.747706	0.0001785	0.815201	0.938008
BAK1	2.553931	3.7116078	0.0002059	1.5565997	4.1902639
MAP3K1	0.5523873	-3.706632	0.00021	0.4035983	0.7560283
NTS	4.6785971	3.6741763	0.0002386	2.0542206	10.655755
LGALS1	1.305645	3.6652875	0.0002471	1.1321114	1.5057784
SORL1	0.6529892	-3.642786	0.0002697	0.5191801	0.8212851
INSR	0.5402697	-3.619063	0.0002957	0.3870807	0.7540842
GPX4	2.111145	3.5979819	0.0003207	1.4052065	3.1717282
MB	8.9376356	3.5806866	0.0003427	2.6949515	29.641101
ITGB2	1.3654905	3.5535211	0.0003801	1.1499249	1.6214662
IL10	1.5850531	3.5385627	0.0004023	1.2281238	2.0457167
SREBF1	1.7737926	3.5329854	0.0004109	1.2906876	2.4377239
ITGAL	1.4785966	3.5277824	0.0004191	1.1898293	1.8374466
BAG3	1.5445637	3.5247225	0.0004239	1.2128807	1.9669511
PRDX5	2.0612662	3.5133581	0.0004425	1.376864	3.0858663
GPX1	1.6735854	3.5104598	0.0004473	1.2553954	2.2310805
UBE2L3	4.7945712	3.5100768	0.000448	1.9981648	11.504513
AIFM1	3.89334	3.4921998	0.0004791	1.8155555	8.3490129
NDUFS8	2.0075078	3.4862155	0.0004899	1.3567573	2.9703823
ECHS1	2.4416478	3.4782874	0.0005046	1.4764859	4.0377249
ABCD1	1.8552749	3.4626999	0.0005348	1.3076253	2.6322869
KCNE1	1.4666302	3.4558844	0.0005485	1.1803039	1.8224155

AKR1B1	1.7381542	3.4517302	0.000557	1.2698756	2.379115
VNN1	1.2337951	3.3831535	0.0007166	1.0924037	1.3934869
HLA-DRB1	1.2926621	3.3814639	0.000721	1.113951	1.5000439
BLOC1S1	1.9644212	3.3758615	0.0007359	1.3273575	2.9072428
HK2	0.5849445	-3.348712	0.0008119	0.4273754	0.8006079
SMAD3	1.7958697	3.3420226	0.0008317	1.2739509	2.5316108
CYC1	2.2909035	3.3269428	0.000878	1.4057888	3.7333053
SRC	1.4836656	3.3255837	0.0008823	1.1758652	1.8720375
SIAH1	0.376497	-3.318313	0.0009056	0.2114388	0.6704066
CYP2E1	0.4096613	-3.307463	0.0009415	0.2414084	0.6951802
MRPS16	3.624904	3.3062035	0.0009457	1.6894201	7.7777747
CTSD	1.3784503	3.2983169	0.0009727	1.1390965	1.6680986
VDR	1.4076807	3.2965425	0.0009788	1.148711	1.7250333
FKBP5	1.4898123	3.2957411	0.0009816	1.17536	1.8883923
ACO2	2.7888158	3.2705458	0.0010734	1.5083072	5.1564386
MAP3K11	2.0997061	3.2612941	0.001109	1.344462	3.2792044
HSD17B10	2.4045841	3.2545968	0.0011355	1.4176608	4.078567
VCL	1.7430168	3.2470574	0.001166	1.2463757	2.4375536
CYP4F2	0.603977	-3.206523	0.0013435	0.443783	0.8219967
PRDX4	1.5943549	3.1972955	0.0013872	1.1978406	2.1221252
BCL2L11	1.6375277	3.1969495	0.0013889	1.2102507	2.2156541
PYCR2	2.7644836	3.1536733	0.0016123	1.4694576	5.2008098
BMP2	2.7259674	3.1489733	0.0016385	1.4603153	5.0885572
TALDO1	2.6366651	3.1469943	0.0016496	1.4415171	4.8226989
CDK6	0.6806561	-3.146919	0.00165	0.5356386	0.8649354
TPI1	2.2747687	3.1462949	0.0016535	1.3632814	3.7956746
HLA-DRA	1.262989	3.140022	0.0016894	1.0917097	1.4611404
ISG15	1.4674494	3.1360069	0.0017127	1.1546845	1.8649319
BCL2A1	1.2719909	3.1114434	0.0018618	1.0931187	1.4801328
THBS1	1.1998644	3.1106277	0.0018669	1.0697248	1.3458364
FADD	2.7333327	3.1058841	0.0018971	1.4491665	5.1554516
SESN1	1.5649476	3.1058471	0.0018973	1.1796672	2.076061
AGRN	0.6997351	-3.086009	0.0020286	0.5577621	0.8778459
LYN	1.6125865	3.0859245	0.0020292	1.1904711	2.1843748
FTH1	1.4939805	3.0552644	0.0022486	1.1547872	1.9328042
CALR	0.6661254	-3.036119	0.0023964	0.5124515	0.8658831
IGF1	62.822996	3.0323276	0.0024268	4.3240763	912.73339
CANX	0.4883124	-3.004738	0.0026581	0.3059413	0.7793947
LDLR	1.4062233	3.0011559	0.0026896	1.1255493	1.7568879
CCL5	1.3947335	3.0001361	0.0026986	1.1222673	1.7333496
PLD1	0.6824231	-2.99746	0.0027224	0.5315518	0.8761165
SLC25A1	1.4757444	2.9851599	0.0028343	1.1429932	1.9053671
RCAN1	1.5071492	2.9851477	0.0028344	1.1512877	1.973007
GSTP1	1.7280786	2.9835859	0.0028489	1.2064361	2.4752703
ECE1	1.4093309	2.982238	0.0028615	1.1248116	1.7658188
KRIT1	0.4679116	-2.977659	0.0029046	0.2838298	0.7713822
GZMB	1.3514163	2.9669137	0.0030081	1.1076152	1.6488812
TGFA	0.5888111	-2.94815	0.0031968	0.4140506	0.8373338
CASP1	1.3555231	2.9397085	0.0032852	1.1066965	1.6602953
COX5A	2.0683266	2.9332296	0.0033546	1.2726957	3.3613494
PECAM1	1.3836557	2.9224267	0.0034732	1.1128733	1.7203243

ANXA11	2.6523514	2.9215216	0.0034833	1.3785715	5.1030855
BRAF	0.4135792	-2.919346	0.0035077	0.2286269	0.7481523
MIR93	0.7059752	-2.908331	0.0036336	0.5583244	0.8926728
HRH1	2.4964946	2.905535	0.0036663	1.3468103	4.6275897
CLIC1	2.0329726	2.9025516	0.0037014	1.259111	3.2824569
MSN	2.4319203	2.8949144	0.0037926	1.3324391	4.4386542
SRXN1	3.1750916	2.8914602	0.0038346	1.4509182	6.9481566
CD4	1.2676311	2.8859888	0.0039019	1.0790641	1.4891503
MRPS34	1.9211259	2.8855816	0.0039069	1.2329832	2.9933294
MIR222	0.6915759	-2.88535	0.0039098	0.5383269	0.8884513
CCL3	1.4354345	2.8846971	0.0039179	1.1228542	1.8350308
MIR25	0.6553981	-2.879624	0.0039815	0.491601	0.873771
UBC	1.8112588	2.8784967	0.0039958	1.2087096	2.7141824
KCNQ1	1.3416572	2.8759304	0.0040284	1.0981283	1.639193
CYB5R3	1.8390091	2.8593995	0.0042444	1.211228	2.7921699
MAPKAPK5	0.3139808	-2.858835	0.004252	0.1419022	0.6947314
TNFRSF1B	1.2116778	2.8390163	0.0045253	1.0612541	1.3834228
NCF2	1.2167643	2.8368845	0.0045556	1.0625234	1.3933955
PARK7	2.4946586	2.8301682	0.0046524	1.3245573	4.6984163
SIRT6	1.9476742	2.8282195	0.0046808	1.2271021	3.0913768
LBR	0.6007412	-2.825332	0.0047232	0.4218523	0.8554889
AREG	1.1520983	2.8175397	0.0048393	1.0440363	1.2713452
PIK3CA	0.4282915	-2.814907	0.0048791	0.2373159	0.7729511
CACNA1C	0.4581209	-2.808917	0.0049708	0.2657198	0.7898348
PF4	1.1655995	2.8006224	0.0051004	1.0470707	1.2975457
IL1RN	1.2683539	2.7999741	0.0051107	1.0739221	1.4979872
CYP3A4	0.0752186	-2.798166	0.0051394	0.0122815	0.4606792
NDUFA6	2.5369807	2.7980586	0.0051411	1.321612	4.8700156
BMP4	0.2905146	-2.795678	0.0051791	0.122127	0.6910737
MYO9A	0.4776259	-2.793961	0.0052067	0.2844251	0.8020617
AIF1	1.4021468	2.782832	0.0053887	1.1051113	1.7790206
HK1	1.5718019	2.7748616	0.0055225	1.1420271	2.1633121
RNF112	16.711702	2.7735961	0.005544	2.2843997	122.25574
ITGAM	1.2244546	2.7721086	0.0055694	1.0611202	1.4129306
ELANE	0.9088407	-2.770016	0.0056054	0.8494057	0.9724344
WRN	0.5568039	-2.76979	0.0056092	0.3679219	0.8426533
CPQ	1.6814733	2.762628	0.0057338	1.1629814	2.4311242
CEBPB	1.3053265	2.7503736	0.0059527	1.0795821	1.5782749
GADD45A	1.660425	2.7483875	0.0059889	1.156575	2.3837721
PFN1	1.7190977	2.7476293	0.0060028	1.1680295	2.5301559
PKM	1.5493002	2.7445117	0.0060601	1.1333193	2.1179656
CUL1	0.584071	-2.733299	0.0062703	0.3971965	0.858867
ACTN4	2.0205886	2.721139	0.0065057	1.2174477	3.3535555
PTGIS	1.4778573	2.7207196	0.006514	1.1154032	1.9580921
NDUFV1	2.0487337	2.7124688	0.0066784	1.2201512	3.4399915
HMOX2	2.5640738	2.7030388	0.0068709	1.2954374	5.0751001
COL2A1	0.547126	-2.702716	0.0068775	0.3533075	0.8472703
ATF2	0.3723482	-2.699506	0.0069443	0.1817351	0.7628859
PXN	1.5177977	2.6948442	0.0070422	1.1205127	2.0559426
NCF4	1.5696905	2.6928967	0.0070834	1.1305642	2.1793795
GLUD2	3.2237212	2.6921512	0.0070993	1.3748601	7.5588624

EDN1	4.5481803	2.6825672	0.0073059	1.5038404	13.755412
G3BP1	0.4382895	-2.680663	0.0073477	0.2397922	0.8011007
MAP2K3	1.8461952	2.6763466	0.007443	1.1783519	2.8925456
MMP7	1.2279733	2.6713073	0.0075556	1.0562097	1.4276695
CXCL10	1.3533763	2.6664271	0.0076662	1.0834753	1.6905115
ALDH3A1	8.1069329	2.6652118	0.007694	1.7397812	37.776223
CDK5	1.9808943	2.6611516	0.0077874	1.1973471	3.277197
XRCC6	2.4229481	2.6539291	0.0079561	1.2603773	4.6578729
VPS13C	0.6302932	-2.621664	0.0087502	0.4463547	0.890031
LMNA	1.2162859	2.6207849	0.0087728	1.0506097	1.4080884
VCP	2.8636885	2.6201989	0.0087879	1.3035739	6.2909449
CCR5	1.2833957	2.615604	0.008907	1.0645405	1.5472446
SYK	1.6499894	2.6154253	0.0089116	1.1337146	2.4013672
PRKCD	1.3830762	2.611369	0.0090181	1.0842614	1.7642423
GSN	1.3966968	2.6019379	0.0092699	1.0859277	1.7964012
MMP2	0.8474372	-2.590519	0.0095831	0.7476775	0.9605075
GFM2	0.4479415	-2.590389	0.0095868	0.2439637	0.8224651
CYP3A5	0.3530911	-2.578647	0.0099188	0.1600468	0.7789805
CX3CR1	1.1524466	2.5733306	0.0100725	1.0343979	1.2839673
SESN2	1.631916	2.5703699	0.010159	1.1233395	2.3707437
SIRT2	2.0802333	2.5652816	0.0103092	1.1886758	3.6404969
MAPK7	1.5559793	2.5584122	0.0105151	1.108951	2.1832089
GSS	2.2343549	2.5516614	0.0107211	1.2049357	4.1432432
OSGIN1	3.4700537	2.5458172	0.0109022	1.3315094	9.0433254
STUB1	1.8556384	2.5441005	0.010956	1.1525167	2.987717
OPTN	1.3408946	2.5296389	0.011418	1.0682903	1.6830615
TRAP1	1.7814055	2.5212483	0.0116939	1.1371695	2.7906177
MATR3	0.4984552	-2.518819	0.0117749	0.2899616	0.8568637
VASP	1.5648632	2.5180949	0.0117992	1.1043464	2.2174173
IGF2R	1.2493343	2.5169803	0.0118365	1.0504982	1.4858056
SCO2	1.4292975	2.5103553	0.012061	1.0814598	1.8890126
NRF1	0.2540152	-2.509881	0.0120772	0.0871194	0.7406351
FH	1.980083	2.5035552	0.0122953	1.1598937	3.3802483
MAPKAPK2	1.5267022	2.4918788	0.0127069	1.094521	2.1295341
CYP19A1	17.482367	2.4854106	0.0129402	1.8310537	166.91655
PTK2	1.2861935	2.4818321	0.0130709	1.0543491	1.5690191
ATXN3	0.4136908	-2.480836	0.0131074	0.2059842	0.8308409
CASP2	0.5675824	-2.47757	0.0132281	0.3626147	0.8884081
TUFM	1.8702452	2.4772532	0.0132398	1.1396636	3.0691662
HSPA1B	1.3953767	2.4760358	0.013285	1.0719081	1.8164581
SERPINE1	0.7762134	-2.466708	0.0136362	0.6346932	0.949289
RHOA	2.0815487	2.4517704	0.0142155	1.1584196	3.740307
TIA1	0.5963609	-2.441129	0.0146414	0.3937923	0.9031319
SCN4A	2.1977683	2.4390292	0.0147268	1.1672687	4.1380235
ABCA1	0.7356364	-2.438157	0.0147624	0.5747482	0.9415617
PIK3CB	0.6386457	-2.437883	0.0147735	0.445345	0.9158481
TRIM21	1.7714815	2.4338034	0.0149411	1.1177608	2.8075297
MMP14	0.8149954	-2.431193	0.0150492	0.6910839	0.9611243
DCTN1	2.5616615	2.4248464	0.0153149	1.1976233	5.4792767
SLC8A1	1.3347604	2.4210977	0.0154737	1.0565375	1.686249
GADD45G	1.8392964	2.416798	0.0156577	1.1220847	3.014934

IGF2BP2	1.3926369	2.4148247	0.0157428	1.0643723	1.8221421
IKBKG	2.1887862	2.4140735	0.0157753	1.1587651	4.1343887
KEAP1	1.7655479	2.4056713	0.0161428	1.111067	2.8055549
EIF2AK3	0.4503817	-2.401888	0.0163107	0.2349088	0.8634998
PIGA	0.4612805	-2.397388	0.0165124	0.2450432	0.8683355
OXA1L	2.5083408	2.3967662	0.0165405	1.1824609	5.3209147
PTK2B	1.7354394	2.3899708	0.0168497	1.104269	2.7273699
MGST1	0.8462967	-2.380271	0.0172999	0.7376381	0.9709614
ATR	0.5450039	-2.379833	0.0173205	0.3306021	0.8984492
PTGS1	1.2943122	2.3746091	0.0175675	1.0460774	1.6014533
MMD	1.5311458	2.3668638	0.0179395	1.0759873	2.178843
CASP9	2.659162	2.3635752	0.0180996	1.1817638	5.9835496
RPS6KB1	0.405708	-2.362894	0.0181329	0.1919712	0.8574149
GLUD1	1.8874103	2.3594328	0.0183029	1.1135408	3.1990903
PGD	1.5671791	2.3251335	0.0200648	1.0731094	2.2887233
ASS1	0.7440977	-2.324028	0.020124	0.5799217	0.954752
GSTO1	1.778663	2.3189663	0.0203969	1.0932446	2.89381
HDAC1	2.4975743	2.3171042	0.0204981	1.151517	5.4170952
HMOX1	1.1809148	2.3143868	0.0206465	1.0257924	1.3594952
LAMP2	0.4894931	-2.310885	0.0208392	0.2670587	0.8971938
PARP1	1.7532239	2.3103385	0.0208694	1.0888779	2.8229007
PDYN	54.265076	2.2918734	0.0219129	1.7831731	1651.3812
FGF7	1.8182287	2.2889417	0.0220827	1.0897276	3.0337451
NEDD8	1.93843	2.2857673	0.0222679	1.0989347	3.4192303
CASP3	0.6673566	-2.282009	0.0224888	0.4715233	0.9445235
KCNE2	6.8844361	2.2803037	0.0225897	1.311308	36.143653
NR1H2	1.7922714	2.2774509	0.0227593	1.0847399	2.9612968
CHAT	0.0004269	-2.276599	0.0228102	5.36E-07	0.3398854
HSF1	1.7784595	2.2688343	0.0232784	1.0815336	2.9244753
FGFR1	0.8160119	-2.263084	0.0236305	0.6842589	0.9731338
IL6R	1.4746814	2.2411539	0.0250161	1.0499436	2.0712399
EIF4EBP1	1.4893081	2.2335549	0.0255124	1.0499995	2.1124187
S100A9	1.0891883	2.2322867	0.025596	1.0104767	1.1740311
ITGB3	1.1978987	2.232072	0.0256102	1.0222569	1.4037188
SMAD4	0.4576459	-2.22702	0.025946	0.2300208	0.9105252
NOS3	2.2449571	2.222094	0.026277	1.1000954	4.5812684
GSTA1	8805.4254	2.2211559	0.0263404	2.9098744	26645657
VWF	1.2055802	2.2074366	0.0272836	1.0211811	1.423277
KCNMA1	2.234207	2.2038338	0.027536	1.0930322	4.5668195
NDUFS6	1.926467	2.2016338	0.0276912	1.0746272	3.4535467
NUDT1	1.6188187	2.2006724	0.0277592	1.0541004	2.4860763
CS	2.9337577	2.1959593	0.0280949	1.1226209	7.6668215
NDUFA12	1.7861998	2.1947462	0.0281818	1.0640208	2.9985407
TTPA	105.20577	2.1932836	0.028287	1.6409784	6744.9113
SLC25A4	2.1911352	2.1840578	0.028958	1.0838124	4.429801
PEPD	1.5754232	2.17042	0.029975	1.0450588	2.3749459
SOD1	1.664627	2.1575069	0.0309662	1.0477651	2.6446606
HTR3A	27.331086	2.157015	0.0310045	1.3528316	552.16648
CYP2C9	1.8969943	2.1554325	0.031128	1.0597827	3.3955899
MIF	1.3889795	2.1548172	0.0311761	1.0301573	1.8727859
CPT2	2.0896763	2.1525246	0.0313561	1.0681534	4.0881269

EPHX1	0.6512962	-2.150938	0.0314811	0.4406473	0.9626448
NDUFB9	1.5896308	2.1480669	0.0317084	1.0414231	2.4264164
GH1	1.7514172	2.1474281	0.0317592	1.05014	2.9210033
BCL2	0.7372981	-2.146708	0.0318165	0.5582132	0.9738367
IRF5	1.3861551	2.1447996	0.0319689	1.0285399	1.8681103
TNIP1	1.8296414	2.1390157	0.0324344	1.0518698	3.1825111
CREB1	0.5334748	-2.12546	0.0335483	0.2988654	0.9522526
GBA	1.6078891	2.1227235	0.033777	1.0370857	2.4928578
TACO1	3.0554253	2.116707	0.0342847	1.0862249	8.5945586
CIITA	1.1931197	2.1097369	0.034881	1.0126139	1.4058019
ACTB	1.5155781	2.1049461	0.035296	1.0290528	2.2321274
S100A8	1.0876281	2.0998123	0.0357454	1.0056101	1.1763356
SOD2	1.3498404	2.096356	0.0360506	1.0197093	1.7868516
PDCD1	1.3152918	2.0933139	0.0363211	1.0176116	1.7000518
STIP1	1.8784893	2.0929264	0.0363557	1.0408662	3.3901784
IDH1	1.5107073	2.090429	0.0365793	1.0260836	2.2242208
COX6B1	1.5125312	2.0815404	0.0373845	1.0244623	2.2331233
MIR199A1	0.734711	-2.081125	0.0374225	0.5495758	0.9822125
STK25	1.8474416	2.0771186	0.0377906	1.0352262	3.2969033
F3	0.803658	-2.076066	0.0378879	0.6538097	0.9878505
APP	0.8920048	-2.072493	0.0382194	0.8006252	0.993814
SLC18A3	3.48E-06	-2.072423	0.038226	2.40E-11	0.5056167
FKBP1B	0.5924393	-2.069667	0.0384835	0.36086	0.9726329
CXCR3	1.3723477	2.0694217	0.0385065	1.0168827	1.8520702
CD86	1.1577936	2.0651408	0.0389097	1.0074899	1.3305206
C5AR1	1.1190228	2.061082	0.0392952	1.0055324	1.2453224
TLR7	1.180608	2.05516	0.0398636	1.0077202	1.3831569
MAPK3	1.6772704	2.052719	0.0400998	1.0236441	2.7482557
ANXA5	1.1441139	2.0510585	0.0402613	1.0059973	1.301193
CYBA	1.3855582	2.0479451	0.0405654	1.0141082	1.8930638
RETN	0.8882834	-2.047022	0.040656	0.7930328	0.9949745
POR	1.5042943	2.0426298	0.0410891	1.0166623	2.2258142
MAP2	9.9557745	2.0325281	0.0421002	1.0855071	91.309806
ACADS	1.6274579	2.0301498	0.0423413	1.0169797	2.6043974
MAP2K1	1.4745236	2.0301297	0.0423434	1.0135122	2.145233
IGF1R	0.8218937	-2.027905	0.0425699	0.679963	0.9934501
SLC7A11	1.5830063	2.0255692	0.042809	1.0149881	2.4689047
CCN2	1.2501801	2.0250889	0.0428582	1.0072066	1.5517673
TAT	488.97531	2.0236902	0.043002	1.2153067	196737.87
PDGFB	1.6027193	2.0134937	0.0440627	1.0126194	2.5366975
SCGB1A1	1.6330735	2.0128654	0.0441288	1.0129736	2.6327725
HSP90B1	0.7383725	-2.003377	0.0451369	0.5487891	0.993449
PGK1	1.8263133	2.0028578	0.0451926	1.0129826	3.2926728
ELAC2	2.1882522	2.0012361	0.045367	1.0162813	4.7117345
PRDX1	1.368804	2.0004143	0.0454555	1.0063683	1.8617681
GNAS	1.990696	1.9949097	0.0460527	1.0121335	3.9153636
MRPS22	0.3870596	-1.994545	0.0460925	0.152301	0.9836778
CD44	0.758864	-1.992323	0.0463356	0.5784612	0.9955283
CNR1	9.0751233	1.9920255	0.0463683	1.0361355	79.485608
GYG1	1.4438975	1.986217	0.0470092	1.0048672	2.0747418
RAD51	1.5949413	1.9835136	0.0473101	1.005558	2.5297772

MIR221	0.7933344	-1.979889	0.047716	0.6308475	0.9976728
CACNB4	0.7748376	-1.977636	0.0479698	0.6017435	0.9977231
TYK2	1.5273489	1.9752992	0.0482342	1.0032935	2.3251367
TSPO	1.291072	1.9751506	0.0482511	1.0019662	1.663596
MDH2	1.74886	1.9715296	0.0486633	1.0032844	3.0484985
PPARD	1.3571931	1.9663456	0.0492587	1.0009917	1.8401481
BAD	1.6341944	1.9642424	0.049502	1.0010704	2.6677358
RPTOR	0.654327	-1.964104	0.049518	0.4285267	0.9991064
