# Supplementary material for: Machine Learning Assistants Construct Oxidative Stress-Related Gene Signature and Discover Potential Therapy Targets for Acute Myeloid Leukemia
Source: Oxid Med Cell Longev. 2022 Aug 22;2022:1507690. doi: 10.1155/2022/1507690 (PMC9423988; doi:10.1155/2022/1507690)
Supplement: Supplementary Materials — Supplement Table 1: prognosis-related oxidative stress genes by filter batch univariate Cox regression. Supplement Table 2: genes with a relative importance of more than 0.3 in the random forest model. Supplement Table 3: different expression genes between low- and high-risk groups. Supplement Table 4: candidate herbs targeting PLA2G4A protein. [file 1507690.f1.zip › 1507690.f2.pdf]

gene	raw.import	rel.importance
PLA2G4A	0.0077795	1
MMP7	0.0054118	0.754386
RCAN1	0.0035515	0.5614035
MB	0.0033824	0.5438596
FKBP5	0.0032133	0.5263158
RYR1	0.0032133	0.5263158
PIK3CA	0.0030441	0.5087719
RAC2	0.0030441	0.5087719
SIGMAR1	0.002875	0.4912281
SOCS1	0.0027059	0.4736842
CHCHD10	0.0025368	0.4561404
ETFB	0.0025368	0.4561404
ATF2	0.0021985	0.4210526
GADD45A	0.0021985	0.4210526
HLA-DRA	0.0021985	0.4210526
INSR	0.0021985	0.4210526
NCF4	0.0021985	0.4210526
CDK5	0.0020294	0.4035088
MIR222	0.0020294	0.4035088
NDUFS2	0.0018603	0.3859649
PTPN1	0.0018603	0.3859649
AGRN	0.0016912	0.3684211
BAK1	0.0016912	0.3684211
G3BP1	0.0016912	0.3684211
GFM2	0.0016912	0.3684211
LDLR	0.0016912	0.3684211
IRAK1	0.0015221	0.3508772
KRIT1	0.0015221	0.3508772
BMP2	0.001353	0.3333333
IL10	0.001353	0.3333333
PARK7	0.001353	0.3333333
MAP2K3	0.0011838	0.3157895
NDUFA6	0.0011838	0.3157895
TPI1	0.0011838	0.3157895
