# Supplementary material for: Machine Learning Assistants Construct Oxidative Stress-Related Gene Signature and Discover Potential Therapy Targets for Acute Myeloid Leukemia
Source: Oxid Med Cell Longev. 2022 Aug 22;2022:1507690. doi: 10.1155/2022/1507690 (PMC9423988; doi:10.1155/2022/1507690)
Supplement: Supplementary Materials — Supplement Table 1: prognosis-related oxidative stress genes by filter batch univariate Cox regression. Supplement Table 2: genes with a relative importance of more than 0.3 in the random forest model. Supplement Table 3: different expression genes between low- and high-risk groups. Supplement Table 4: candidate herbs targeting PLA2G4A protein. [file 1507690.f1.zip › 1507690.f3.pdf]

gene	lowMean	highMean	logFC	pValue	fdr
TRH	3.8141753	1.1835981	-1.688192	5.52E-11	4.59E-07
HOXA7	0.6919737	1.9192487	1.4717526	1.30E-09	1.35E-06
HOXA6	1.1823426	3.2846928	1.4741103	1.46E-09	1.40E-06
HOXA-AS3	0.5930467	1.7305136	1.5449826	1.80E-09	1.40E-06
LINC02593	2.1253003	0.797087	-1.414858	1.84E-09	1.40E-06
HOXA-AS2	0.760569	2.1014862	1.466259	2.41E-09	1.48E-06
HOXA3	0.9968046	2.7248471	1.4507926	2.67E-09	1.53E-06
HOXA4	0.7464388	2.0677508	1.4699665	2.96E-09	1.64E-06
LPO	3.4987313	1.6107271	-1.11912	3.54E-09	1.83E-06
AL645608.2	2.4426054	1.0302287	-1.245456	3.65E-09	1.83E-06
AL645608.6	2.626094	1.0990491	-1.256663	4.43E-09	2.11E-06
HOXA5	1.6275831	4.0376989	1.3108022	6.55E-09	2.67E-06
HOXA9	1.8287143	4.2471047	1.21565	6.74E-09	2.67E-06
METTL7B	1.3182481	2.8250411	1.09965	7.33E-09	2.83E-06
SAMD11	1.624803	0.6392778	-1.34575	1.34E-08	3.98E-06
PRRT4	2.6011782	1.1215701	-1.213645	1.34E-08	3.98E-06
HOXA10-AS	1.0540058	2.408859	1.1924671	1.34E-08	3.98E-06
CPNE8	1.446598	3.2444311	1.1653015	1.86E-08	5.03E-06
HOXA10	1.3451308	3.0799706	1.1951701	2.40E-08	6.11E-06
IL2RA	0.9135024	1.9897766	1.1231261	2.03E-07	3.12E-05
LDLRAD3	0.9611128	2.2485429	1.2262128	3.42E-07	4.47E-05
HNMT	0.8340728	1.9860742	1.2516743	3.59E-07	4.62E-05
NKX2-3	0.6714594	1.8469154	1.4597458	5.12E-07	5.91E-05
SHD	1.5744593	0.6414256	-1.295503	6.67E-07	7.01E-05
AC005392.2	0.7265714	1.7183366	1.2418362	1.16E-06	0.0001076
THBS1	1.3855993	2.8274581	1.0289955	1.31E-06	0.000114
AC111000.4	2.2947896	0.9220513	-1.315443	1.69E-06	0.0001346
TMEM273	0.9813928	2.0272705	1.046636	1.74E-06	0.0001365
LINC01475	0.7897509	1.9469623	1.3017554	1.93E-06	0.0001463
UGT2B11	2.0715502	0.8089454	-1.356597	1.98E-06	0.0001471
PDCD6IPP1	0.6114728	1.7467153	1.5142843	4.20E-06	0.0002365
HOXB6	0.7327363	2.0563458	1.4887169	4.49E-06	0.0002453
HOXB5	0.7880666	2.03172	1.3663122	7.37E-06	0.0003407
KCNE1	0.6395998	1.3942035	1.1241997	7.65E-06	0.0003471
AL513542.1	0.7388463	1.7769084	1.2660232	1.40E-05	0.0005031
HOXB-AS3	0.7784259	1.9515713	1.3260045	4.17E-05	0.0010648
BEX2	0.726045	1.5243547	1.0700678	5.10E-05	0.0012044
TSPAN7	1.7830866	0.8390001	-1.087634	6.21E-05	0.0013562
CLEC4O	3.9894868	1.5832842	-1.333283	6.55E-05	0.0014114
AC103702.1	0.6839345	1.5150299	1.1474163	7.65E-05	0.0015694
AC112482.1	3.0737618	1.454644	-1.079339	9.04E-05	0.0017637
RNU6-446P	1.4491846	0.6736465	-1.105178	0.0001124	0.0020616
AC087623.1	1.4048694	0.637123	-1.140792	0.0001244	0.0022084
UGT3A2	1.8423424	0.6268709	-1.555301	0.0001745	0.0028246
RP11-79902	2.0937286	0.8713882	-1.264687	0.0002517	0.0035899
IL12A-AS1	0.6990743	1.4845539	1.0865117	0.0003307	0.0042454
AL136313.1	1.5896234	0.7445021	-1.094337	0.0007317	0.0072336
AC005336.3	2.0385135	0.9888677	-1.043668	0.0013464	0.0111866
CES1	0.7603687	1.755338	1.2069778	0.0045696	0.0250283
