# Supplementary material for: Machine Learning Assistants Construct Oxidative Stress-Related Gene Signature and Discover Potential Therapy Targets for Acute Myeloid Leukemia
Source: Oxid Med Cell Longev. 2022 Aug 22;2022:1507690. doi: 10.1155/2022/1507690 (PMC9423988; doi:10.1155/2022/1507690)
Supplement: Supplementary Materials — Supplement Table 1: prognosis-related oxidative stress genes by filter batch univariate Cox regression. Supplement Table 2: genes with a relative importance of more than 0.3 in the random forest model. Supplement Table 3: different expression genes between low- and high-risk groups. Supplement Table 4: candidate herbs targeting PLA2G4A protein. [file 1507690.f1.zip › 1507690.f4.pdf]

Herb id	Pinyin nam	Latin name	English na	Class in E	P_value	FDR(BH)	Relationsh
SMHB00146	Guadi	Calycis Me	Muskmelon	Emetic Med	7.00E-06	0.000217	By_ingredi
SMHB00190	Huomaren	Cannabis F	Hemp Seed	Laxatives	0.000986	0.007157	By_ingredi
SMHB00102	Difuzi	Kochiae Fr	fruit of E	Diuretic D	0.000599	0.011309	By_ingredi
SMHB00485	Zhiqiao	Aurantii F	fruit of S	Qi Regulat	0.00433	0.017769	By_ingredi
SMHB00258	Lulutong	Liquidamba	fruit of t	Wind-Dampn	0.004839	0.01876	By_ingredi
SMHB00181	Huajuhong	Citri Gran	Pummelo Pe	Qi Regulat	0.001555	0.020506	By_ingredi
SMHB00399	Tianguazi	Melo Semen	muskmelon	Phlegresol	0.006363	0.021326	By_ingredi
SMHB00323	Qiegeng	Radix Sola	root of Ga	Wind-Dampn	0.008646	0.024939	By_ingredi
SMHB00391	Tiandong	Asparagi R	Cochinchin	Yin-Tonify	0.009152	0.025632	By_ingredi
SMHB00008	Baqia	Smilacis C	Smilax Chi	Wind-Dampn	0.010671	0.027527	By_ingredi
SMHB00252	Longkui	None	Solanum Ni	Antipyreti	0.011177	0.02822	By_ingredi
SMHB00330	Qingpi	Citri Reti	Green Tang	Qi Regulat	0.01143	0.028568	By_ingredi
SMHB00124	Fenbixie	Dioscoreae	Dioscoreae	Diuretic D	0.011683	0.028888	By_ingredi
SMHB00246	Liangtouji	Anemones R	Radde Anem	Wind-Dampn	0.011683	0.028888	By_ingredi
SMHB00373	Shishangba	None	Selaginell	Antipyreti	0.012188	0.029535	By_ingredi
SMHB00256	Luhui	Aloe	Aloe	Offensive	0.003093	0.029905	By_ingredi
SMHB00456	Yimucao	Leonuri He	motherwort	Blood Acti	0.012946	0.030654	By_ingredi
SMHB00043	Bichengqie	Litseae Fr	Litseae Fr	Warming In	0.013198	0.030921	By_ingredi
SMHB00185	Huangjing	Polygonati	Rhizome of	Yin-Tonify	0.013198	0.030921	By_ingredi
SMHB00282	Mianbixie	Dioscoreae	Dioscoreae	Diuretic D	0.01446	0.032752	By_ingredi
SMHB00062	Cheqianzi	Plantagini	seed of As	Diuretic D	0.015217	0.033625	By_ingredi
SMHB00312	Puhuang	Typhae Pol	pollen of	Stasis-Res	0.015217	0.033625	By_ingredi
SMHB00379	Shuifeiji	Silybi Fru	Silybum Ma	Antipyreti	0.015217	0.033625	By_ingredi
SMHB00324	Qinjiao	Gentianae	root of La	Wind-Dampn	0.015469	0.033958	By_ingredi
SMHB00375	Shijunzi	Quisqualis	fruit of R	Antiparasi	0.015469	0.033958	By_ingredi
SMHB00486	Zhishi	Aurantii F	immature f	Qi Regulat	0.015469	0.033958	By_ingredi
SMHB00279	Meihua	Mume Flos	Plum Flowe	Qi Regulat	0.005121	0.036489	By_ingredi
SMHB00030	Baiziren	Platycladi	seed of Ch	Tranguiliz	0.017735	0.036579	By_ingredi
SMHB00177	Huzhang	Polygoni C	rhizome of	Diuretic D	0.017986	0.036897	By_ingredi
SMHB00189	Huangyaozi	Rhizoma Di	rhizome of	Phlegresol	0.018489	0.037495	By_ingredi
SMHB00405	Tufuling	Smilacis G	Glabrous G	Antipyreti	0.01874	0.037817	By_ingredi
SMHB00268	Machixian	Portulacae	all-grass	Antipyreti	0.019494	0.038594	By_ingredi
SMHB00482	Zhimu	Anemarrhen	rhizome of	Fire Purgi	0.019996	0.03926	By_ingredi
SMHB00645	Huangshany	Dioscorea	None	Qi Regulat	0.006431	0.039939	By_ingredi
SMHB00064	Chenpi	Citri Reti	Dried Tang	Qi Regulat	0.021	0.0405	By_ingredi
SMHB00232	Kunbu	Laminariae	Kelp or Ta	Phlegresol	0.021753	0.041292	By_ingredi
SMHB00498	Ziwan	Asteris Ra	root of ta	Antitussiv	0.023006	0.042865	By_ingredi
SMHB00272	Maiya	Hordei Fru	Germited B	Digestants	0.024007	0.044027	By_ingredi
SMHB00298	nshetenggu	None	Celastri C	Tranguiliz	0.026007	0.046359	By_ingredi
SMHB00557	Dilong	Pheretima	None	Liver-Paci	0.010165	0.047557	By_ingredi
SMHB00174	Huluba	Trigonella	Trigonella	Yang Reinf	0.028503	0.04928	By_ingredi

ip
ent
ent
ent
ent
ent
ent
ent
ent
ent
ent
ent
ent
ent
ent
ent
ent
ent
ent
ent
ent
ent
ent
ent
ent
ent
ent
ent
ent
ent
ent
ent
ent
ent
ent
ent
ent
ent
ent
ent
ent
ent
